# BEstimate: a computational tool for the design and interpretation of CRISPR base editing experiments

**DOI:** 10.1186/s13059-026-04077-z

**Published:** 2026-04-27

**Authors:** Cansu Dinçer, Bo Fussing, Mathew J. Garnett, Matthew A. Coelho

**Affiliations:** 1 Somatic Genomics Programme, Wellcome Sanger Institute, Hinxton, Cambridgeshire UK; 2 Cellular Informatics, Informatics and Digital Solutions, Wellcome Sanger Institute, Hinxton, Cambridgeshire UK

**Keywords:** Base editing, CRISPR, guideRNA, Library design, Screening

## Abstract

CRISPR base editors enable scalable targeted DNA mutagenesis and are a powerful tool for analysing the function of variants of uncertain significance and disease modelling. Existing guide RNA (gRNA) design tools lack comprehensive functional annotation of target sequences. Here we developed BEstimate, a flexible computational pipeline that systematically specifies base editor gRNA target sites, generates on-target activity and off-target predictions, and provides functional, structural and clinical annotations of installed variants. BEstimate supports custom gRNA design against variant alleles and reversion of disease variants. BEstimate is a freely available, versatile tool for designing gRNA libraries and analysing base editor screens.

**Supplementary Information:**

The online version contains supplementary material available at 10.1186/s13059-026-04077-z.

## Background

Genome editing has the potential to inform gene and gene variant function at scale [1, 2] with implications for our understanding and treatment of genetic diseases [3–6]. Specifically, clustered regularly interspaced short palindromic repeats (CRISPR) base editing enables the programmable introduction of single-nucleotide variants (SNVs) without generating DNA double-strand breaks [7–9]. Base editors offer scalable, affordable, multiplexed genome editing in a range of cell types [10, 11]. This technology has broad applications in both basic and translational research, such as functional genomics and therapeutic development. Example applications include studying variant effects on the cytotoxic functionality of primary human T cells and pinpointing mutations that tune immune responses [12]. Similarly, *JAK1* mutagenesis with base editing predicted variants altering IFN-γ pathway activity in colorectal cancer cells [13], and base editing screens elucidated variants related to sensitivity or resistance to cancer therapies [14]. Furthermore, base editing screening of *WRN* confirmed the helicase domain as the primary therapeutic target in microsatellite-unstable (MSI) cancer cell lines [15]. For therapeutic purposes, base editors have been successfully used to correct disease-causing mutations in sickle cell disease, β-thalassemia and Alpha-1 Antitrypsin Deficiency [16–18].

Base editors comprise a catalytically impaired Cas9 (typically Cas9 nickase or dead Cas9/dCas9) and a nucleotide deaminase. Using a dCas9 provides recognition of the targeted sequence via gRNAs without causing double-strand breaks, which can be deleterious in some cells. By overcoming the requirement for Homology-Directed Repair (HDR)-dependency and insertion and deletion (indel) formation, base editors can generate mutations at the targeted site through deamination [19]. Base editors facilitate the precise installation of SNVs in a defined editing window within the genomic gRNA target site. There are several types of base editors with different specificities, enabling a range of edits to be introduced for each nucleotide position. Cytosine base editors (CBEs) use cytosine deaminase fused to Cas9 nickase leading to transition from cytosine to thymine (C > T) [9, 20]; adenine base editors (ABEs) use adenine deaminase leading to an adenine to guanine (A > G) transition [8], and lastly glycosylase base editors (CGBEs) employ both cytosine deaminase and uracil-DNA glycosylase for cytosine to guanine (C > G) transversions [21, 22].

Base editors cannot target all positions on the genome due to the limitation of protospacer adjacent motif (PAM) preference [8, 9], activity window restriction, and the nature of the deamination reaction [23]. Moreover, editing efficiency varies depending on sequence context [24, 25] and on Cas9 protein variant and positional preferences [24, 26]. Therefore, generating the intended alteration with high efficiency depends on the model, base editor, and gRNA sequence, and necessitates experimental optimisation [24]. Further important considerations in base editing gRNA design include potential off-target and bystander mutations [23, 27, 28]. Technological improvements have led to base editors with less stringent PAM requirements, reduced off-targets, more precise editing windows, and different nucleotide conversions, such as cytosine to guanine [26, 29–33]. Furthermore, information about the potential functional consequences of the generated alterations allows experiment-specific library design and in silico analysis of the sequence of interest. In addition to selecting favourable gRNAs with minimal off-target effects and maximum on-target efficiency, it can be useful to prioritise programmed DNA variants based on their potential functional, clinical, and structural consequences. It is therefore vital to have a robust and flexible tool that provides customisation options for Cas9 and base editor enzymes, on- and off-target information, and annotation of mutational consequences.

Current computational tools can identify either gRNAs targeting a given DNA sequence [34–37] or gRNAs to introduce a specific alteration, for example generating a disease-associated variant [34–36] (Additional file 1: Table S1). However, most existing tools have limited flexibility in nuclease, PAM, and activity window selection [38] and can only accommodate currently available base editors, such as SpCas9 NGG. Importantly, they lack a comprehensive annotation of predicted edits. Thus, we developed BEstimate, a Python package that systematically analyses gRNA target sites across reference or variant sequences for fully customised base editors, PAM, and activity windows. BEstimate comprehensively annotates functional, structural and clinical consequences of programmed edits and provides information on gRNA on-target and off-target scores. This pipeline identifies editable positions or alterations that can be correctable by base editors before starting experiments. To facilitate broad accessibility and rapid exploration of annotated gRNA libraries, BEstimate is complemented by an online resource (bestimate.sanger.ac.uk) that provides immediate access to precomputed datasets for 50 representative highly mutated cancer genes. Together, because of its flexible design and detailed multi-level annotations, BEstimate provides insight ranging from gene sequence to predicted clinical consequences of base editing and offers a framework that is readily extendable to accommodate future improvements in base editing enzymes [39].

## Results

**Fig. 1:**
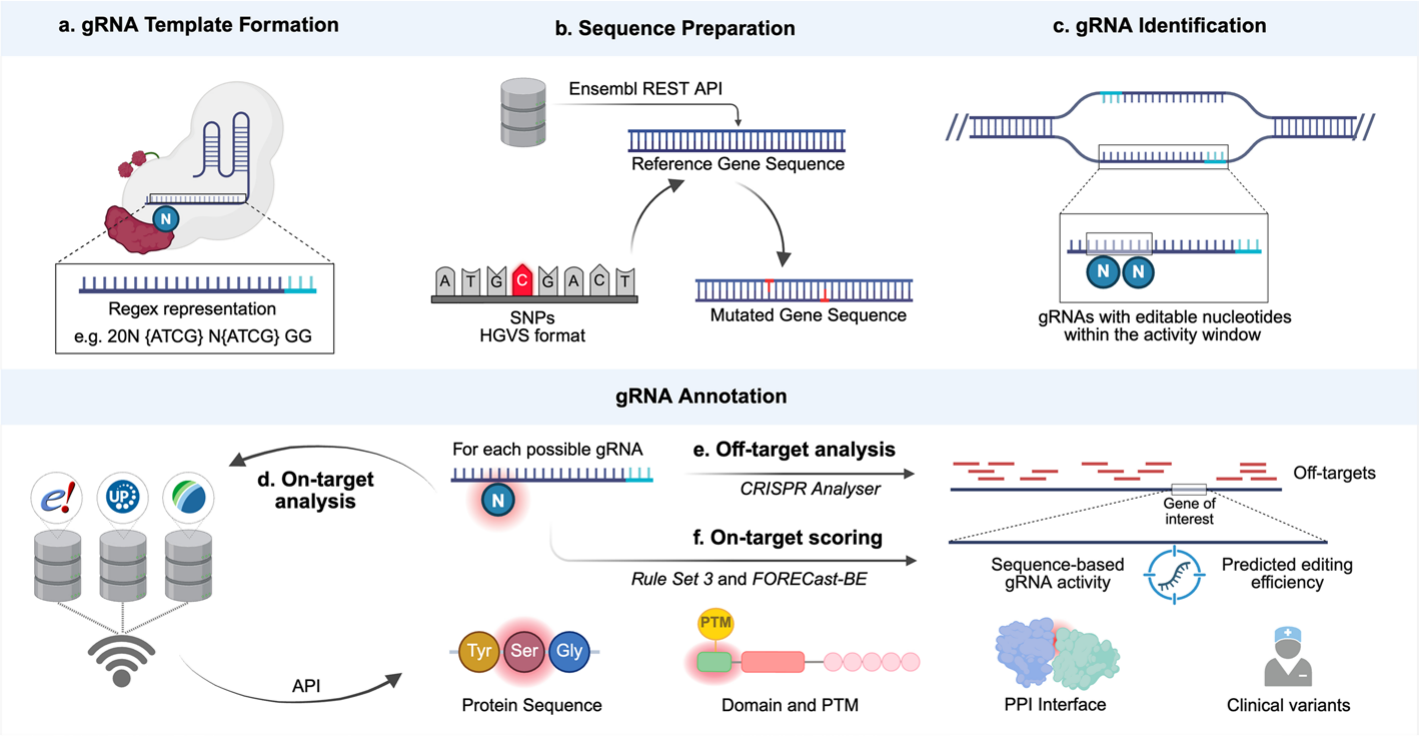
BEstimate is a flexible tool for gRNA identification and annotation in base editing experiments. Given a user-defined input, BEstimate (**a**) designs a regular expression (regex) representation of the gRNA, (**b**) retrieves the reference gene sequence and if provided, prepares the altered gene sequence, (**c**) searches sites across the reference or altered gene sequence, aligning with the gRNA regex representation and finds genomic coordinates of gRNA and editable sites, (**d**) annotates on-target functional, structural and clinical consequences of potential gRNAs, (**e**) predicts gRNA off-target scores across the reference genome, and (**f**) calculates on-target gRNA activity scores and predicts on-target base editing efficacy

We have previously used a prototype version of BEstimate to design three independent gRNA libraries collectively targeting 20 genes [13–15]. Here, BEstimate is implemented as a Python package retrieving real-time, up-to-date information with *Ensembl* Application Programming Interface (API) [40, 41] and *Uniprot* Representational State Transfer (REST) API [42, 43] to collect and manipulate gene sequences, identify gRNA sequences for fully customisable base editors and comprehensively annotate them with functional, structural and clinical consequences (Fig. 1). Here we demonstrate the use of BEstimate through three case studies, each highlighting different functions of the tool.

To exemplify the capabilities of BEstimate, we also benchmarked BEstimate to another tool, BEscreen [38], a recently developed base editor gRNA design tool that has input flexibility and VEP annotation implementation. We performed comparisons for on-target gRNA identification and on- and off-target analysis of *KRAS* in Case Study 1, gRNA annotation with *MYC* in Case Study 2, and SNP-based queries against the *HBB* gene in Case Study 3.

### Case study I: designing base editor libraries with reference and model-specific gene sequences

A key application of base editing is the analysis of variants involved in cancer and cancer drug resistance [14]. To facilitate this and to demonstrate the utility of BEstimate, and extending previous gRNA libraries from BEstimate [13–15], we designed a cytosine and adenine NGG- and NG-PAM-specific base editor gRNA libraries targeting 50 frequently mutated cancer genes derived from the *cBioPortal* pan-cancer study (bestimate.sanger.ac.uk) [44, 45]. To highlight the base editor coverage across nucleotide and amino acid sequences, we leveraged BEstimate for in silico analysis, collecting editable sites and consequences of the generated alterations on the genome and translated proteins.

**Fig. 2:**
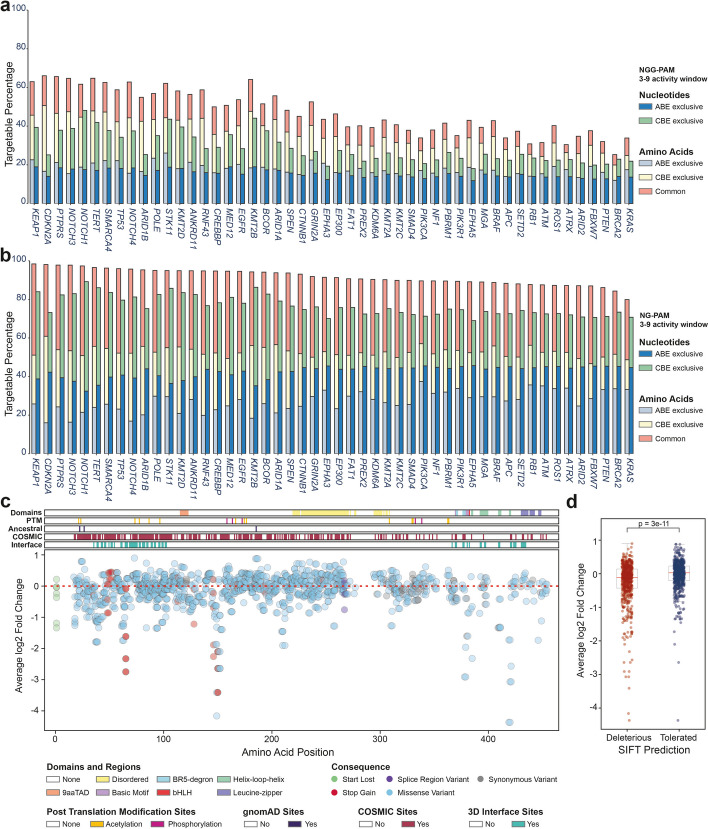
BEstimate gRNA design and in silico annotation. **a** and **b** Barplot of (**a**) NGG-PAM specific cytosine (CBE) and adenosine (ABE) base editor coverage and (**b**) NG-PAM CBE and ABE base editor coverage at the nucleotide and amino acid level across 50 cancer genes, indicated in the x-axis. The y-axis shows the percentage of targetable nucleotides and amino acids. When an amino acid can be targeted only with one base editor, it was assigned as exclusive to the corresponding base editor; otherwise, it was labelled as commonly targeted. **c** Average log2 fold change (LFC) (versus the plasmid library control) of *MYC* base editor screens with functional annotation of the alterations. The colour of dots represents the predicted mutational consequence of the gRNA edit. The top annotations indicate domains and regions, post-translational modification sites, and ancestral sites whose positions are in the *Genome Aggregation Database* *(gnomAD)*, the *Catalogue of Somatic Mutations in Cancer* *(COSMIC)*, or on protein–protein interaction (PPI) sites from Interactome Insider, respectively from top to bottom. **d** Average *MYC* log2 fold change comparison between gRNAs predicted as deleterious and tolerated by the Sorting Intolerant From Tolerant (SIFT) algorithm. The boxplot shows the values between the 25th and 75th quantiles, with the red line indicating the median. The *p*-value indicates the significance based on a two-way Mann–Whitney U Test

For the selected 50 genes with NGG-PAM and 3–9 activity window, we covered an average of 13.15% of nucleotide positions (standard deviation (σ) 5.76%) for cytosine, and 15.39% of nucleotide positions (σ = 2.01%) for adenine base editors (Fig. 2a). Leveraging a less stringent NG-PAM, there was an increase in the average editable nucleotide coverage to 34.11% (σ = 8.22%) for cytosine, and 42.54% (σ = 3.20%) for adenine base editors (Fig. 2b).

At the amino acid level, synonymous mutations (base editors combined, NGG-PAM mean = 14.76%, σ = 3.86% and NG-PAM mean = 14.80%, σ = 3.73%) were rarer than nonsynonymous ones (base editors combined, NGG-PAM mean = 85.24%, σ = 3.86% and NG-PAM mean = 85.21%, σ = 3.73%) across 50 genes analysed. Amongst nonsynonymous mutations, gRNAs most frequently generated missense mutations both in NGG-PAM (base editors combined, mean = 84.27%, σ = 7.17%) and NG-PAM (base editors combined, mean = 84.10%, σ = 7.04%) specificities, followed by splice-site-affecting changes (base editors combined, NGG-PAM mean = 8.60%, σ = 3.81% and NG-PAM mean = 8.64%, σ = 3.61%). While ABE cannot generate nonsense mutations, nonsense mutations introduced by CBE were more frequent than splice-site mutations (NGG-PAM mean = 9.06%, σ = 3.00% and NG-PAM mean = 9.53%, σ = 2.73%).

To highlight gRNAs with functional consequences, we only considered gRNAs which can generate nonsynonymous mutations, and we observed a coverage of the NGG-PAM library of 29.36% (σ = 10.12%) for cytosine, and 29.10% (σ = 6.03%) for adenine base editors (Fig. 2a). Similar to the nucleotide coverage, NG-PAM specific base editors increased the amino acid coverage of cytosine and adenine base editors to 64.66% (σ = 8.58%) and 66.09% (σ = 4.10%), respectively (Fig. 2b). Across all editable amino acids, 11.24% (σ = 3.98%) and 38.99% (σ = 3.75%) of them were shared between both base editors with NGG- and NG-PAM specificities, respectively (Fig. 2a, b). Using both base editors combined, the predicted average edited amino acid coverage per gene increased to 47.22% (σ = 11.57%) for NGG and 91.76% (σ = 4.01%) for NG PAM-specific base editors with a 3–9 activity window. These results demonstrate the value of using multiple base editors with permissive PAM sequences to increase coverage of amino acid positions and achieve a high overall saturation.

To compare the outputs of BEstimate with those of the BEscreen webserver, we identified *KRAS* gRNAs with NGG-based ABE (activity window: 3–9). BEstimate recovered all 43 on-target gRNAs reported by BEscreen and additionally identified 357 (291 without Poly-T RNA polymerase III termination signals) gRNAs that were not returned by BEscreen. These gRNAs mapped to exonic, non-coding regions, outside the coding sequence (CDS) (Additional file 1: Table S2). The BEscreen webserver offers gRNA identification only within CDS regions with an option to add splice sites, whereas BEstimate performs gRNA identification across the full gene sequence and annotates each gRNA with genomic location and sequence-level properties. This strategy allows users to flexibly filter gRNAs, for example by restricting analyses to CDS regions or by applying sequence constraints such as GC content or poly-T motifs.

We further evaluated the on- and off-target gRNA predictions of the tools across the gRNAs identified by both tools (Additional file 1: Table S2). BEscreen does not provide an explicit on-target activity score; instead, it reports positional features based on distance from the centre of the activity window. In contrast, BEstimate measures both gRNA activity using Rule Set 3 [46] and predicted editing efficiency using FORECast-BE [25], enabling the ranking and prioritisation of gRNAs.

BEscreen identifies potential off-target sites using a BLAST-based approach, reporting exact sequence matches across the genome. BEstimate performs a comprehensive genome-wide off-target search, finding both exact matches and off-targets containing up to four mismatches with off-target counts stratified by each mismatch level. Due to the methodological differences, we only compared off-target scores with exact matches and found discrepancies in off-target counts for the 3 gRNAs due to BEstimate querying the protospacer with the PAM sequence (not just the protospacer), which is required for editing. Additionally, the additional mismatch-level resolution provided by BEstimate offers expanded flexibility for off-target assessment, which is highly relevant for base editing experiments where mismatched sites can still be susceptible to unintended edits.

Existing variants in target genes may negatively impact gRNA efficiency or create de novo PAM sequences. As a unique property, we designed BEstimate to generate gRNAs that account for SNVs within genes, including disease-associated variants. For example, we designed an NG-PAM-specific ABE gRNA library for the *PIK3CA* gene specific to the head and neck cancer cell line, CAL-33 (Additional file 1: Table S3). CAL-33 has a *PIK3CA* missense mutation (g.179234297A > G in GRCh38, p.His1047Arg). BEstimate gRNA design enabled the identification of a new gRNAs due to the generation of a novel PAM with the mutated G nucleotide (TGAAACAAATGAATGATGCACGT, GRCh38 genomic location: 179,234,276–179,234,298 on chromosome 3) and editable sites (GRCh38 genomic location: 179,234,278, 179,234,279, 179,234,280, 179,234,282, 179,234,283, 179,234,284, on chromosome 3, details in Additional file 1: Table S3). The library with the reference sequence prevents the manipulation of the 179,234,282 genomic location, whereas the CAL-33 specific sequences allow ABE to generate a missense mutation, p.Gln1042Arg, within the catalytic domain in the *PIK3CA*-encoded p110α subunit.

In summary, BEstimate can design base editor gRNAs for both reference and altered gene sequences, enabling maximal flexibility in both base editor properties and reference sequence, as well as providing on- and off-target scores, allowing further prioritisation of gRNAs.

### Case study II: functional annotation of a *MYC* base editor screen

BEstimate utilises the *Ensembl* REST-API to use *Ensembl* Variant Effect Predictor (VEP) to collect variant consequences [40, 47]. To illustrate the in silico annotation functionality, we obtained data from *MYC* ABE and CBE base editor fitness screens in the colon cancer cell line HT-29 and annotated the gRNA effects [14] (Additional file 1: Table S4). The transcription factor MYC is an oncogene and is an essential gene in HT-29 cells [48]. We linked the potential consequences from VEP to the measured viability changes. As expected, while synonymous mutations did not cause a reduction in cell viability, highly depleted gRNAs were predicted to cause missense and nonsense mutations (Fig. 2c). While 73% of all designed gRNAs were predicted to generate at least one alteration reported in the *Catalogue Of Somatic Mutations In Cancer (COSMIC)* [49], 61 gRNAs were predicted to alter a post translational modification sites (acetylation sites at amino acid position 158, 163, 172, 332 and 338, and phosphorylation sites at positions 21, 23, 73, 77, 86, 96, 166, 174, 176, 308, 329, 330, 362 and 363) (Fig. 2c). Additionally, we identified 10 gRNAs predicted to generate mutations overlapping human genetic variation sites (Fig. 2c), retrieved from the *Genome Aggregation Database (gnomAD)* [50]. Naturally occurring variants can potentially lead to different effects across populations; thus, this result underscores the importance of cautious interpretation, especially in clinical applications.

MYC binds DNA through heterodimerisation with MAX [51–53]. Blocking MYC-MAX protein interactions negatively affects cancer cell growth [54–57]. Concordantly, BEstimate indicates that the highly depleted gRNA (GACGGACAGGATGTATGCTGTGG for ABE, GRCh38 genomic location: 127,740,834–127740856 on chromosome 8, p.Ser420Pro, Additional file 1: Table S4) results in a missense mutation, adding proline, on the helix-loop-helix motif (Log2 Fold Change- LFC = −4.367) at the interface between MYC and MAX (Fig. 2c). Moreover, BEstimate further collects predicted variant consequences from various tools such as Sorting Intolerant From Tolerant (SIFT) [58], Polymorphism Phenotyping (PolyPhen) [59] and Combined Annotation Dependent Depletion (CADD) [60] via the *Ensembl* VEP API. From the collected information from these tools, we facilitated categorical SIFT prediction of *MYC* mutagenesis to demonstrate that tolerant and deleterious SIFT categories broadly differentiated the differences in *MYC* depletion (two-sided Mann–Whitney U Test, *p*-value = 3e-11, Fig. 2d), with some exceptions, highlighting the importance of experimental data from these screens.

When we retrieved the *MYC* gRNAs and annotations from BEscreen, we found similar VEP outputs, including translational consequences, functional, clinical, and population-level effects of the potential edits. However, BEstimate additionally provided affected PTM and PPI sites, and thus, depletion of a gRNA introducing a proline on the MYC-MAX interface can only be found using BEstimate. In summary, BEstimate integrates functional and structural interpretation to elucidate mechanisms of variant effects.

### Case study III: identifying gRNAs to correct a sickle cell disease-causing variant

In addition to the functional analysis of alterations in wild-type genomic sequences, base editors are being explored for therapeutic genome editing, including correction of disease-associated pathogenic mutations and other clinically relevant applications [16, 17, 61–63]. There are examples of clinical-stage studies in which disease-causing alterations can be corrected with base editing, such as in sickle cell disease, β-thalassemia [16, 17] and Alpha-1 Antitrypsin Deficiency [18]. In addition to enabling gRNA design to target SNVs, BEstimate can identify gRNAs predicted to revert disease-associated alterations. As an example, we retrieved the β-globin gene (*HBB*) missense mutation (g.5227002A > T in GRCh38, p.Glu7Val, rs334) causing sickle cell disease [64], and identified an NG-PAM-specific gRNA for ABE (ACTTCTCCACAGGAGTCAGATGC, GRCh38 genomic location: 5,226,994–5,227,016 on chromosome 11). This gRNA enables the mutant codon GTG (valine) to be changed to GCG (alanine), generating the non-sickling variant, Makassar haemoglobin (Additional file 2: Fig. S1) [16, 65]. This capability could be extended to other monogenic diseases that could be amenable to therapeutic genome editing. Despite identifying gRNAs capable of generating the specified variant, BEscreen could not provide gRNAs to correct the variant because it only works from reference sequences. However, the SNP-aware functionality of BEstimate discovers gRNAs which can revert the given variant within the mutant sequence context, providing a unique feature.

## Discussion

The broad applications of base editors require robust gRNA library design tools with flexible utility and comprehensive annotations for different experimental purposes. Limitations of current tools include restrictive user-defined parameters, a lack of SNP-aware mode and limited annotation of the effects of programmed variants. In contrast, BEstimate offers full customisation, accommodating all current and future base editor enzymes and Cas9 proteins while incorporating on- and off-target annotations and extensive up-to-date functional, structural, and clinical annotations of predicted edits on genes and proteins. Generating non-reference sequences with the provided list of alterations, BEstimate can generate gRNAs targeting mutant genes, including disease-associated variants and SNPs in genomes of individuals from diverse ancestries. By using APIs, BEstimate ensures up-to-date real-time data retrieval, eliminating the need for manual sequence collection and minimising potential errors. In addition to designing libraries for reference gene sequences, BEstimate can also identify gRNAs for mutant gene sequences and design gRNAs to revert pathogenic variants.

To highlight the functionality of BEstimate, we designed gRNA libraries using various PAM sequences and base editors and explored their coverage and predicted effects across 50 cancer genes. Due to the inherent constraints of base editors, which require specific PAM sequences and at least one editable nucleotide within a defined activity window, the number of editable sites across the genome is constrained. Expanding base editing capabilities with Cas9 variants with less stringent PAM specificity and different base editors targeting different nucleotides can improve coverage across both nucleotide and amino acid sequences. BEstimate allows users to easily generate gRNA libraries tailored to different editing constraints, such as PAM sequence flexibility, editable window size, and editor type, enhancing coverage at both the nucleotide and amino acid levels.

Cellular models used in base editing experiments might intrinsically harbour genomic alterations that affect the presence or absence of gRNA-editable sites. Additionally, these alterations can lead to disease, and base editors can be leveraged to convert them into a non-disease genotype. The SNP-aware functionality of BEstimate enables the design of gRNAs not only for reference genomes but also for variant sequences, making it highly suitable for disease models and personalised editing strategies. BEstimate can identify gRNAs either to introduce functional mutations for screening purposes or to design gRNAs capable of reverting disease-causing variants. The flexibility, precision, and extensive annotation make BEstimate a powerful tool for diverse applications, including functional genomics and therapeutic genome editing.

Base editors induce precise genomic changes, which might have significant consequences. Annotating the functional, structural and clinical implications of potential edits helps to interpret base editing experiment results and to link genomic changes to phenotypic outcomes. The *MYC* screen analysis in this study demonstrated the utility of BEstimate by identifying gRNAs predicted to induce deleterious mutations, impact post-translational modifications, or affect functional domains and protein interaction interfaces. While in silico annotation can reveal the functional consequences of experiments, it can also aid in designing base editing libraries before the experiment, focusing on edits with specific predicted functional outcomes. This comprehensive annotation property distinguishes BEstimate from previous tools by providing a more informative approach to interpreting results and strategically designing targeted edits.

Depending on the targeted sequence and its context, base editing can have different efficacies. Integration of Rule Set 3 and FORECast-BE scoring into BEstimate allowed systematic prioritisation of gRNAs by combining predicted activity and expected editing efficiency. Additionally, by integrating genome-wide off-target identification and reporting off-target burden, BEstimate enables users to evaluate potential risks alongside predicted on-target efficiency. This combined view complements genomic and functional annotations by providing quantitative measures that support the selection of gRNAs, and enables guide prioritisation, which is particularly important for multiplexed and therapeutic applications.

Together, BEstimate extends existing base editing gRNA design tools by integrating gRNA identification across the entire gene, variant generation and reversion analyses with SNP-aware mode, expanded functional and structural annotation, and comprehensive on- and off-target evaluation. BEstimate serves as one of the most comprehensive tools in terms of gRNA annotation, covering sequence-level consequences, structural context, and clinical and population-level variant information, influencing experimental design and interpretation.

BEstimate currently retrieves gene sequences exclusively from the human genome within the *Ensembl* database. Future enhancements could include expanding the compatibility of BEstimate with additional genomes and genomic regions. Moreover, tools enabling the integration of three-dimensional locations of editable positions within protein structures will further extend the application for drug design and protein engineering [66, 67].

In summary, BEstimate provides a systematic approach for the flexible design and annotation of gRNA libraries for base editing mutagenesis screens and therapeutic applications.

## Conclusions

In this study, we present BEstimate, a versatile Python module that provides a comprehensive approach for designing and annotating base editor gRNA libraries, providing functionality beyond existing tools. Full customisation on defining base editor and gene sequences, integration of *Ensembl* and *Uniprot* APIs for real-time data retrieval, dual functionality on reference and altered sequences, and extensive annotations, including on-target scoring and off-target prediction, make BEstimate a valuable tool for diverse research applications. To further improve accessibility, we also provide a website (bestimate.sanger.ac.uk) hosting fully annotated, precomputed gRNA libraries for the 50 cancer genes across different base editors and PAM specificities, enabling rapid exploration of BEstimate outputs without local computation, alongside an open-source command-line tool that supports genome-wide and user-defined analyses.

## Methods

### Overview of BEstimate workflow

BEstimate is a Python-based command line tool (see all inputs in the GitHub page in detail: https://github.com/Garnett-Lab/BEstimate) to find and annotate Base Editor gRNAs in 6 steps.

#### Step 1—gRNA pattern formation

For any type of Base Editor, BEstimate can design a representative template via regular expression using user input as the target and edit nucleotides, protospacer length, PAM specificity and location according to the protospacer, and activity window indices on the protospacer (Fig. 1a).

#### Step 2—sequence preparation

BEstimate can retrieve the sequence of the given HUGO symbol through *Ensembl* REST-API [47] from the user-defined *Ensembl* genome assembly [41] (Fig. 1b). In the case where the user may want to integrate one or several SNPs, BEstimate can alter the gene sequences. The SNP should be in the Human Genome Variation Society (HGVS) format [68]. After the sequence is prepared, BEstimate generates two sequences for right (5’ to 3’) and left (3’ to 5’) directions, allowing it to find gRNAs in both directions.

#### Step 3—gRNA identification

BEstimate uses the generated regular expression pattern to search the prepared sequences in both directions. The pattern is scanned through the sequence, and when a match is identified, the start position index and direction are stored (output file: crispr_df). It then uses the base editor specification from the user to select gRNAs having at least one editable nucleotide inside the defined activity window (Fig. 1c). BEstimate then collects *Ensembl* Transcript, Exon and Protein IDs if gRNAs or editable nucleotides are found, as well as labels gRNAs containing poly-T motifs and reports GC content (output file: edit_df). When mutations are provided, BEstimate additionally annotates whether a mutation occurs within the PAM sequence, generates a novel PAM, is located within the gRNA sequence, or is revertible by the specified base editor.

#### Step 4—gRNA annotation

The gRNAs can be annotated functionally, clinically and structurally (Fig. 1d). BEstimate utilises *Ensembl* REST-API [40] to access *Ensembl* [41] and *Ensembl* VEP [47] data to find genomic, transcriptomic and proteomic positions of the potential edits. If an *Ensembl* Transcript ID is not provided, BEstimate uses the canonical transcript selected with Matched Annotation from *NCBI* and EMBL-EBI (*MANE*) [69] with a *RefSeq* [70] match. To retrieve the effects of potential edits with the gRNAs, BEstimate first prepares the Sequence Variant Nomenclature in HGVS [68] of all single and multiple edits (output file: hgvs df). For single edits, substitution versions of HGVSs are prepared; for multiple edits, BEstimate uses indel nomenclature by deleting the reference and inserting the altered activity window sites of the gRNAs. Then, it utilises HGVS symbols as inputs to VEP API to collect information on regulatory regions, DNA motifs and bound transcription factors, mutational consequences, locations of the alterations on genome, cDNA and protein sequences, corresponding *Uniprot* accession [43], predicted and clinical effects of the alterations and corresponding *Clinvar* [71] and *COSMIC* [49] IDs, and lastly ancestral allele for the given variants from *gnomAD* [72]. For the protein level, BEstimate also collects positions of domains and post-translational modification sites (phosphorylation, ubiquitination, methylation and acetylation) via *Uniprot* API [42] after aligning the *Ensembl* Protein and *Uniprot* indices. If *Ensembl* API is not providing the index mapping, BEstimate manually maps those two sequences (Python BioPython package [73]) and provides the alignment table as a Comma-Separated Values (CSV) file. These indices are used to label alterations at protein–protein interaction interface sites, which were collected from the *Interactome Insider* database [74]. BEstimate, thus, maps the positions of corresponding proteins with the genome positions. It therefore merges nucleotide alterations onto protein consequences (output file: protein_df). To ease analysis, BEstimate further summarises all annotations, with each row representing a unique gRNA and each annotation element merged into a semicolumn (output file: summary_df).

#### Step 5—off-target identification and scoring

BEstimate facilitates the Python implementation derived from the CRISPR-Analyser framework from the Wellcome Sanger Institute Cellular Informatics Group [75]. For off-target analysis, the framework first constructs a genome-wide index of gRNA target sites using reference genome assemblies obtained from *Ensembl*. Indexing is performed in three steps: first, scanning genomic FASTA files to identify all candidate gRNA sites matching the specified PAM pattern; second, encoding these gRNA sequences in a compact binary format; and third, storing gRNA metadata in an embedded SQLite database. The gRNA sites are identified by sliding a fixed-length window (size: 23) corresponding to the protospacer (size: 20) and PAM length (size: 3) across the genome. PAM sequences are detected on both forward (5′−3′) and reverse-complement strands by matching the PAM and its reverse complement. For each identified gRNA, the framework records the chromosome, genomic start position, full nucleotide sequence including PAM, and orientation. To efficiently search off-targets, gRNA sequences are encoded using a 2-bit representation per nucleotide (A = 00, C = 01, G = 10, T = 11). PAM orientation is encoded as an additional binary flag. These representations are stored in an indexed array and cross-linked to detailed gRNA metadata stored in the SQLite database. After preparing the binary file and the gRNA metadata, off-target sites for identified gRNAs are queried against the binary index using bitwise comparison operations. Both forward and reverse-complement representations of the gRNA are evaluated to account for PAM orientation. The framework identifies exact matches and off-targets with up to 4 mismatches via the Hamming distance. Off-target counts are reported stratified by mismatch level (0–4), allowing users to assess off-target burden across different thresholds. These summaries are provided alongside gRNA annotations (output file: ot_annotated_df).

#### Step 6—on-target scoring

For all identified gRNAs, BEstimate computes on-target activity and predicts editing outcomes using Rule Set 3 [46] and FORECast-BE [25], respectively (Fig. 1f). To calculate Rule Set 3, first flanking sequences from 3’ and 5’ ends of gRNA were collected through *Ensembl* REST-API. Then, Rule Set 3 scores for predicted sequence-dependent activity are obtained for both tracrRNA variants, Hsu2013 and Chen2013. Additionally, FORECast-BE is used to model base editing outcome distributions for each gRNA based on the sequence context surrounding the target site. BEstimate only collects the average expected editing efficiency score of nucleotides between 3–9 from a protospacer length of 20. On-target scoring can be added with and without gRNA annotation (output file: scored_df).

### Output interpretation

BEstimate generates several output files that integrate gRNA sequence features, functional and structural annotations of potential editing outcomes, off-target burden and predicted on-target activity and editing efficiency.

The first file generated is ‘crispr_df’, which includes all gRNA sequences that meet the PAM requirements of the given base editor. BEstimate later controls whether gRNAs include editable nucleotides within the given activity window, and stores them in the ‘edit_df’ file.

If the annotation option is activated, BEstimate collects functional interpretations, describing predicted coding consequences (e.g. synonymous, missense, nonsense), deleteriousness scores (SIFT, PolyPhen, CADD), structural context (protein domains, post-translational modification sites and protein–protein interaction interfaces) and clinical relevance where available, and stores them in the ‘protein_df’ file. Moreover, Bestimate summarises these annotations in the ‘summary_df’ file, where each row corresponds to a gRNA.

When off-target analysis is selected, BEstimate annotates ‘summary_df’ with off-target counts stratified by mismatch level and creates an ‘ot_annotated_df’ file. Additionally, when activated, BEstimate derives on-target activity scores using Rule Set 3, predicts editing efficiency using FORECast-BE, and further annotates the ‘scored_df’ file.

Collectively, these outputs allow flexible guide selection depending on experimental objectives, such as maximising loss-of-function edits and on-target scores, targeting disease-associated variants, or minimising off-target effects. A detailed description of output fields and example workflows is provided in the BEstimate GitHub documentation.

### Designing base editor libraries for 50 frequently mutated genes across pancancer

*cBioPortal* [45] was used to retrieve 50 frequently mutated genes with nucleotide lengths under 500,000 base pairs from a pan-cancer study comprising 25,000 patients [44] (Additional file 1: Table S5). Two NG- and NGG-PAM-specific gRNA libraries were designed, and in silico annotation was applied to 50 genes using default parameters, except for the activity window, which was set to 3–9.

### Designing a base editor library for the CAL-51 cell line-specific *PIK3CA* gene

A nonsynonymous single-nucleotide alteration in *PIK3CA* in the head and neck cancer cell line CAL-33 was obtained from the *Cell Model Passport* [76]. A mutation file with its HGVS symbol (3:g.179234297A > G) was created. Then, BEstimate was used to generate a *PIK3CA* library specific to the CAL-33 cancer cell line using default settings except for the PAM sequence (NG) and the activity window (3-9) and to annotate the library with functional, structural, clinical, and off-target information.

### Identification of gRNAs correcting sickle cell disease-associated *HBB* mutation

The genomic location of the missense mutation causing Sickle cell disease [64] was retrieved from *dbSNP* (rs334) [77]. The collected mutation was used to generate mutation files with HGVS symbols from genomic position and nucleotide changes. BEstimate was run for the *HBB* gene for NG-PAM-specific adenine base editor (activity window: 3–9) with the prepared mutation file. The gRNAs annotated with ‘guide_change_mutation’ in the edit_df file were collected as sequences targeting and manipulating the mutated nucleotide.

### In silico annotation of the* MYC* base editing screen

The results of *MYC* ABE and CBE screenings were collected from a study within the group [14]. The in silico annotations of the gRNAs were identified with BEstimate using the VEP functionality leveraging the *Ensembl* VEP REST API [40, 47].

### Collecting outputs from the BEscreen web server

Gene-level analysis to identify gRNAs and their provided annotations was performed for the *KRAS* and *MYC* genes using the BEscreen web server [38]. Additionally, a variant-level analysis was performed for the rs334 variant on the *HBB* gene. To allow comparison with BEstimate, the GRCh38 *Homo Sapiens* genome (version 112) was used. For all analyses, only MANE transcripts were enabled. For *KRAS* searches, an NGG-Cas9-based ABE, for *MYC* and *HBB* genes, an NG-Cas9-based ABE were used (activity window: 3–9). *Ensembl* VEP annotations and genome-wide BLAST analyses were performed for the main and mitochondrial chromosomes. Lastly, the expanded version of all outputs were collected.

## Supplementary Information

Additional file 1: Excel document containing suppmentary tables. Table S1. Comparison with the previously designed tools. Table S2. gRNA-level comparison of BEstimate and BEscreen with *KRAS* gene. Table S3. gRNA library for CAL-51-specific *PIK3CA* gene. Table S4. In silico annotation of the *MYC* gene. Table S5. The 50 selected frequently altered genes and their lengths.

Additional file 2: PDF document containing the supplementary figure. Fig. S1. Conversion of sickle cell variant into Makassar, non-sickling variant.

## Data Availability

The source code is freely available at https://github.com/Garnett-Lab/BEstimate [78] under the AGPLv3 licence, and deposited in Zenodo via the following DOI: 10.5281/zenodo.18386204 [79]. Project name: BEstimate. Project home page: https://github.com/Garnett-Lab/BEstimate. Archived version: BEstimate v.1.2.0, https://doi.org/10.5281/zenodo.18386204. Operating system: Linux x86_64. Programming language: Python. License: AGPLv3. The Jupyter Notebooks used to generate the results are archived on Zenodo and can be accessed via the following DOI: 10.5281/zenodo.18245772 [80]. All data generated or analysed during this study are included in this published article and its additional files, which are available in the figshare repository with the identifier 10.6084/m9.figshare.28582844 [81] for additional files and 10.6084/m9.figshare.28582916 [82] for the additional figure. Precomputed and fully annotated gRNA libraries for 50 cancer genes are hosted in the BEstimate website (https://bestimate.sanger.ac.uk/).
