# Supplementary material for: BEstimate: a computational tool for the design and interpretation of CRISPR base editing experiments
Source: Genome Biol. 2026 Apr 27;27:191. doi: 10.1186/s13059-026-04077-z (PMC13262425; doi:10.1186/s13059-026-04077-z)
Supplement: Supplementary file 2 — Additional file 2: PDF document containing the supplementary figure. Fig. S1. Conversion of sickle cell variant into Makassar, non-sickling variant. [file 13059_2026_4077_MOESM2_ESM.pdf]

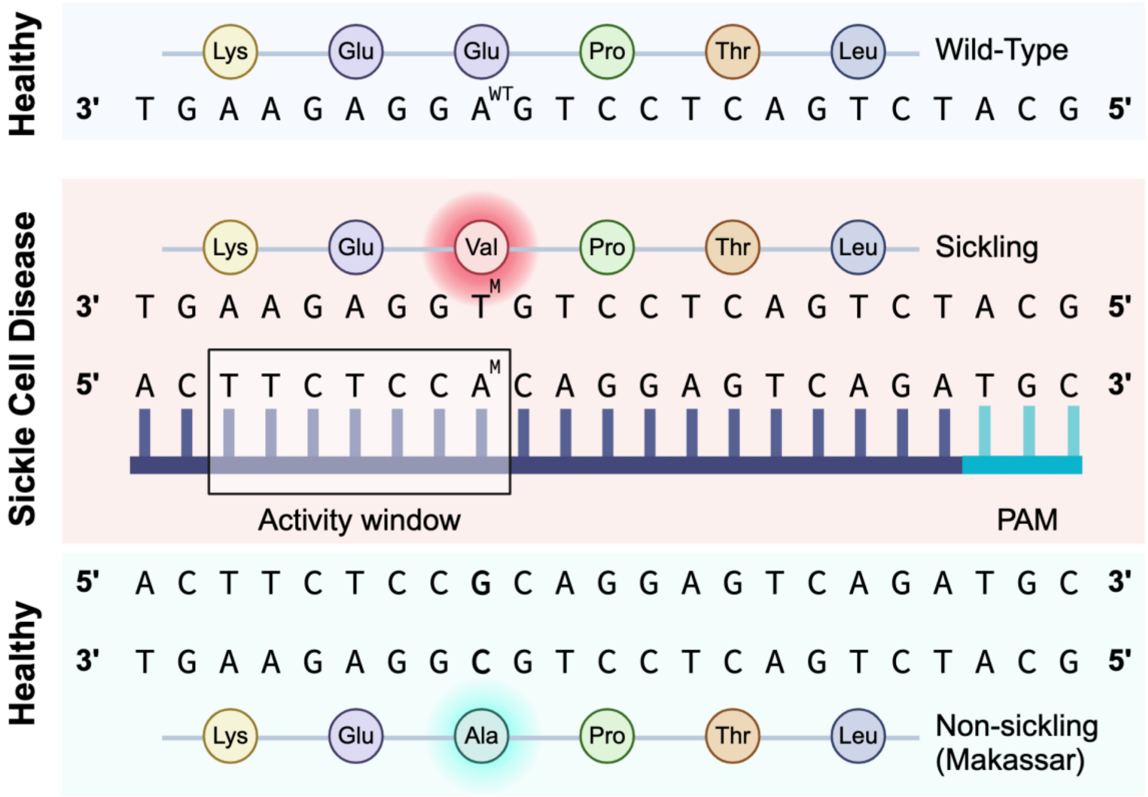

Fig S1: Conversion of sickle cell variant into Makassar, non-sickling variant.

Both nucleotide and corresponding amino acid sequences of β-globin (*HBB*) gene in heathy and sickle cell contexts were indicated with an ABE NG-PAM specific gRNA sequence, generating the non-sickling healthy variant from the sickle cell sequence.
